# Predicting Microstructural Void Nucleation in Discontinuous Fiber Composites through Coupled in-situ X-ray Tomography Experiments and Simulations

**DOI:** 10.1038/s41598-020-60368-w

**Published:** 2020-02-27

**Authors:** Imad Hanhan, Ronald F. Agyei, Xianghui Xiao, Michael D. Sangid

**Affiliations:** 1 School of Aeronautics and Astronautics, Purdue University, West Lafayette, IN 47907 USA; 2 Advanced Photon Source, Argonne National Laboratory, Lemont, IL 60439 USA; 3 Present Address: National Synchrotron Light Source II, Brookhaven National Laboratory, Upton, NY 11973 USA

**Keywords:** Aerospace engineering, Mechanical engineering, Structural materials

## Abstract

Composite materials have become widely used in engineering applications, in order to reduce the overall weight of structures while retaining their required strength. In this work, a composite material consisting of discontinuous glass fibers in a polypropylene matrix is studied at the microstructural level through coupled experiments and simulations, in order to uncover the mechanisms that cause damage to initiate in the microstructure under macroscopic tension. Specifically, we show how hydrostatic stresses in the matrix can be used as a metric to explain and predict the exact location of microvoid nucleation that occurs during damage initiation within the composite’s microstructure. Furthermore, this work provides evidence that hydrostatic stresses in the matrix can lead to coupled microvoid nucleation and early fiber breakage, and that small fragments of fibers can play an important role in the process of microvoid nucleation. These results significantly improve our understanding of the mechanics that drive the initiation of damage in the complex microstructures of discontinuous fiber reinforced thermoplastics, while also allowing scientists and engineers to predict the microstructural damage behavior of these composites at sub-fiber resolution and with high accuracy.

## Introduction

Composite materials have gained attention in many engineering applications, especially in the aerospace and automotive industries due to their low weight and high strength. Specifically, polymer matrix composites have allowed for major weight savings and higher performance aircraft and vehicles. Despite their high rate of implementation, scientists and engineers have faced challenges in predicting their mechanical behavior and performance, especially past the small strain regime and into the damage initiation and damage propagation regimes, because there exist a number of damage mechanisms which are often coupled and are active throughout the life of a component. Compared to continuous fiber composites, discontinuous fiber reinforced polymers exhibit vastly heterogeneous microstructures that can vary significantly depending on the component geometry, making mechanical behavior predictions even more complicated^1–3^.

Until recently, most efforts in predicting the mechanical performance of discontinuous fiber composites have been focused on the elastic loading regime during which damage has not yet initiated and progressed^4,5^. Efforts in understanding and predicting the behavior of fiber composites past the elastic regime have typically focused on attempting to replicate the macroscopic bulk stress-strain behavior of a specimen through phenomenological damage parameters^6^. In recent years, some microstructural approaches have been used to explore the damage mechanisms in certain composites using homogenization approaches^7^ as well as unit cell methods^8^. Experimentally, one *in-situ* study of a thermoset polymer, reinforced with discontinuous carbon fibers, showed that fiber tips play an important role in microvoid nucleation due to high shear stresses, while fiber breakage plays no role^9^. However, for thermoplastic polymers reinforced with discontinuous fibers, researchers have faced even more difficulty because compared to thermoset polymers, thermoplastic polymers experience highly non-linear plasticity creating more complications from a microstructural point of view, in which researchers have applied orientation averaging in unit cells to model this behavior^10^.

Efforts in experimentally examining the damage mechanisms of short fiber reinforced thermoplastics through *in-situ* scanning electron microscopy have shown that under tensile loads, damage initiation appears in the form of microvoid nucleation at fiber tips^11,12^. In attempting to improve predictive capabilities and include this damage initiation mechanism, researchers have modeled the macroscopic behavior of short fiber reinforced thermoplastic composites by defining fiber matrix debonding at fiber tips as the governing damage mechanism with good results at simulating the macroscopic stress-strain response^13^. To try and explain this phenomena, researchers have hypothesized that the underlying mechanism of microvoid nucleation in a thermoplastic matrix is similar to that of ductile metals and is related to high tensile hydrostatic stresses or high stress triaxiality^14–16^. However, the appropriate treatment of microvoid nucleation in composites still remains uncertain, mainly because of its stochastic nature and the challenges in computing the local stress states in complex composite microstructures^17^.

**Figure 1:**
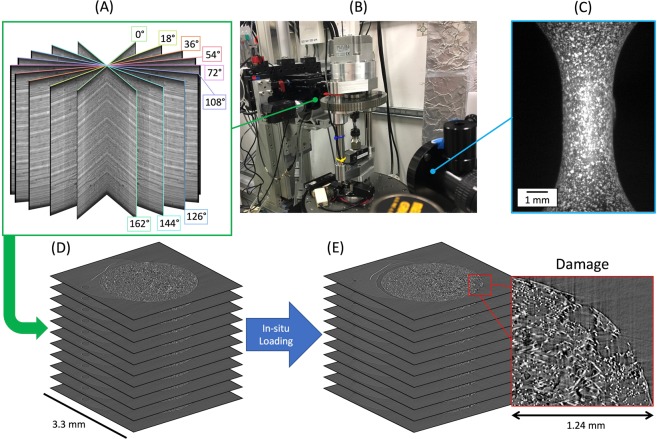
An overview of the *in-situ* study where (**A**) shows a sample 9 of the 1500 X-ray projected radiographs captured by the detector over 180$^\circ$, (**B**) shows the *in-situ* loading set-up with the miniature load frame, (**C**) shows the view of the speckled specimen from the optical camera used for computing macroscopic strain, and (**D** and **E**) show the reconstructed tomography images at 0 load and at maximum load (just before fracture), respectively.

Recently, high resolution X-ray micro computed tomography ($\mu$-CT) has become a popular tool to characterize composite materials’ 3D microstructural features^18–22^, sometimes *in-situ*, in order to observe the evolution of the microstructure^9,23,24^. In this work, high resolution X-ray $\mu$-CT was conducted *in-situ* (with a pixel size of 1.3 $\mu m$) in order to study damage initiation events within the microstructure of a discontinuous glass fiber reinforced polypropylene specimen as shown in Fig. 1, by conducting digital volume correlation (DVC) of the 3D tomography images, and post-processing the tomography images to extract all the microstructural features. The resultant complex 3D microstructure was then examined during deformation and associated initiation of microstructural damage, while a virtual representation of the exact microstructure was simultaneously simulated to quantify the local stresses and strains using a finite element model, including non-linear plasticity in the thermoplastic matrix as well as any porosity manufacturing defects. Through the coupled experiments and simulation of the exact microstructure, this work provides evidence that microvoid nucleation during damage initiation in a fiber reinforced thermoplastic is hydrostatic stress based, validating and propelling forward engineers’ and scientists’ predictive capabilities past the elastic regime and towards the strength prediction of complex heterogeneous composites.

## Results

### The role of porosity manufacturing defects and the ductile fracture zone

**Figure 2:**
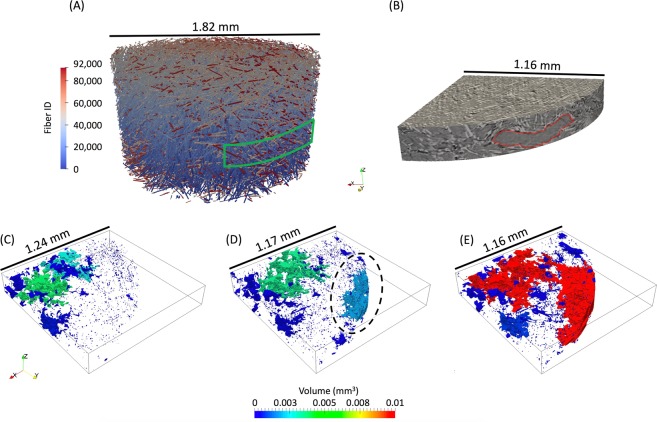
Shown in (**A**) are the fibers detected within the gauge section of the specimen at 0 load where each fiber is individually detected and assigned a fiber ID, and where the green outline indicates the location of ductile fracture observed experimentally, which is also shown in the tomography images just before fracture in (**B**). Preexisting porosity, microvoid nucleation, and eventual void coalescence in the ductile fracture zone are shown in (**C**) at 0 load, (**D**) at 96% of failure, and (**E**) just before failure.

The specimen was loaded in tension until fracture, and the *in-situ* X-ray $\mu$-CT images allowed for the observation of the fibers, porosity manufacturing defects, as well as the tracking of microstructural damage events that led to final fracture. Each fiber detected in the cylindrical specimen is shown in Fig. 2A, with a green outline highlighting the region of the specimen which experienced ductile fracture that led to final fracture, shown in Fig. 2B. Manufacturing defects in the form of preexisting large pores were detected in the center of the specimen as can be seen in Fig. 2C, with preexisting small pores present closer to the free surface. As the specimen was loaded and ductile fracture occurred, it was found that these large pores did not significantly coalesce and grow leading to final fracture as expected in typical ductile fracture^25^. Instead, a large void nucleation event occurred near the free surface of the specimen circled in Fig. 2D, which - just before fracture - finally coalesced with the preexisting large pores leading to final fracture shown in Fig. 2E. This shows that while porosity manufacturing defects have historically been viewed as a major detriment to a composite’s strength^26^, in this discontinuous reinforced thermoplastic, a variety of complex microstructural features exist which can be more detrimental to the overall mechanical performance. In this composite specimen, it was found that these features existed at the critical region near the free surface circled in Fig. 2D, which was analyzed in more detail to detect the exact locations of damage initiation, and simulated through a finite element model to compute the stresses and strains.

### Experimental microvoid nucleation during damage initiation

**Figure 3:**
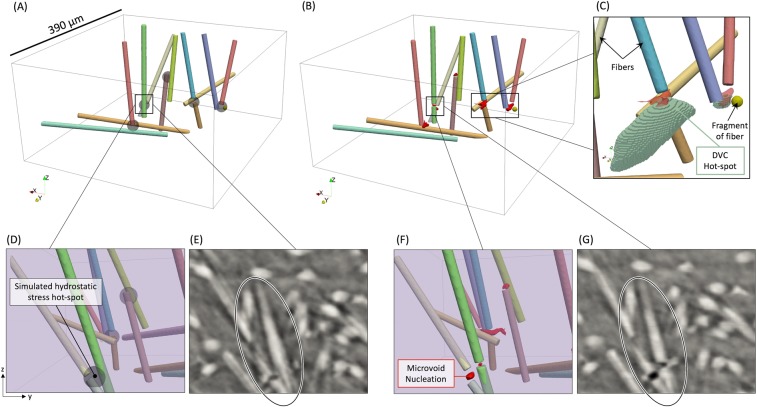
The fibers that interacted with the microvoid nucleation events (**A**) at the unloaded state with grey spheres at the locations of simulated matrix hydrostatic stress hot-spots, and (**B**) at 50% of the macroscopic failure strain with the experimentally determined microvoid nucleation shown in red, with (**C**) an overlay of a sample of the DVC ${\varepsilon }_{zz}$ strain hot-spots. The coupled microvoid nucleation and early fiber breakage is shown in (**D** and **E**) at the unloaded state, and in (**F** and **G**) at 50% of the macroscopic failure strain.

Experimentally, five locations within the critical region showed evidence of microvoid nucleation during damage initiation and have been visualized at the unloaded state in Fig. 3A and at 50% of the macroscopic failure strain in Fig. 3B, with a sample of the DVC results overlaid in Fig. 3C. Two of the five locations consisted of T-type intersections, where at least one fiber was well aligned with the loading direction and interacted with fiber(s) that were highly misaligned with the loading direction. One region consisted of two well aligned fibers with slightly overlapping end-points, where a microvoid nucleated at one fiber end-point, but not both. Another region consisted of two fibers highly aligned with the loading direction that had nearly touching end-points, which resulted in microvoid nucleation between the end-points and a coupled early fiber breakage in a neighboring fiber shown in Fig. 3D–G. Lastly, one case of microvoid nucleation consisted of two well aligned fibers with end-points near a small fragment of glass fiber, all of which are shown in Fig. 3B. It is important to note that the locations of microvoid nucleation, while always relating to fiber tips, were not confined to one single configuration of fiber tips, but instead included a few different configurations.

### Simulated heterogeneous stresses and strains in the microstructure

**Figure 4:**
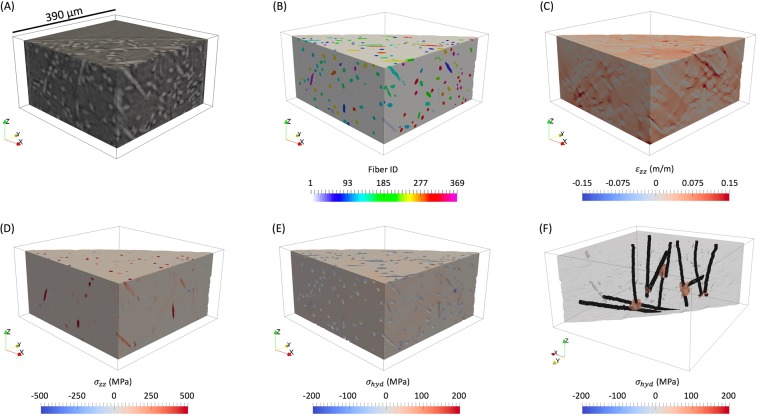
An overview of the cropped simulation results (cropped to visualize away from the boundary conditions) of the specimen region shown in the tomography images of (**A**) which were post-processed for feature detection shown in (**B**). The heterogeneous strain in the loading direction (${\varepsilon }_{zz}$) is shown in (**C**), the heterogeneous stress in the loading direction (${\sigma }_{zz}$) is shown in (**D**), the heterogeneous hydrostatic stress (${\sigma }_{hyd}$) of the matrix is shown in (**E**), and the agglomerations of matrix elements in the $99.99{7}^{\mathrm{th}}$ percentile of hydrostatic stress, with the fibers they interact with, are shown in (**F**).

The results of the finite element simulation of the exact microstructure experimentally analyzed, which has been visualized in Fig. 4A,B, showed that there was in fact a large degree of heterogeneity in the strains and stresses. As expected, the ductile thermoplastic matrix experienced most of the deformation as can be seen by the strain plot in Fig. 4C, while the fibers, acting as the toughening constituent in the composite, experienced most of the stress in the specimen, shown by the stress plot in Fig. 4D. The hydrostatic stress was computed by

1 $${\sigma }_{hyd}=\frac{{\sigma }_{xx}+{\sigma }_{yy}+{\sigma }_{zz}}{3}$$

and was found to be normally distributed in the matrix, visualized in Fig. 4E, with an average hydrostatic stress of 10.83 MPa and a standard deviation of 15.96 MPa. While there were scattered regions within the matrix whose elements experienced high hydrostatic stress (sometimes in a single line of elements and other times isolated only to the side of a fiber at a corner of a 3D voxel), only large agglomerations, referred to as hydrostatic stress hot-spots, were of interest because they ensured that the region of high ($99.99{7}^{\mathrm{th}}$ percentile) matrix hydrostatic stress was not artificially induced from meshing effects, and are shown in Fig. 4F.

## Discussion

The matrix hydrostatic stress hot-spots computed in the simulation have been overlaid onto the experimental results in Fig. 3A as transparent black spheres within the microstructure. These simulated hydrostatic stress hot-spots correlated directly to the five locations of microvoid nucleation experimentally observed in the microstructure during damage initiation, shown by the red regions in Fig. 3B. Of the five cases of hydrostatic stress hot-spots in the matrix and their corresponding microvoid nucleation events, two were of interest because of their unique behavior. The first case of interest was a location that experienced coupled microvoid nucleation and fiber breakage. Fiber breakage occurring at 50% of the macroscopic failure strain of 5.77%, experimentally observed in Fig. 3D–G, is atypical in these composites^11^. Specifically, the green fiber shown first at the unloaded state in Fig. 3D, and then at 50% of the failure strain in Fig. 3F, was the only fiber that broke in the critical region at this early loading condition; yet this fiber was simulated to experience (prior to microvoid nucleation) only 45% of its ultimate tensile strength^27^ at the same loading condition observed experimentally. When overlaying the simulation results, it can be seen that a matrix hydrostatic stress hot-spot was in contact with the fiber at the location of fiber breakage, which experimentally resulted in microvoid nucleation in the matrix next to the fiber, followed by early fiber breakage shown in Fig. 3E,G. This provides evidence that high hydrostatic stress can be used not only as a metric to describe and predict microvoid nucleation during damage initiation, but also - in certain cases - describe microvoid nucleation coupled with other damage mechanisms.

Another case of microvoid nucleation that was of interest consisted of two fibers that were well aligned with the loading direction which interacted with a small fragment of a glass fiber that was approximately spherical, which was also experimentally verified by the DVC analysis shown in Fig. 3C. The experimentally-based DVC analysis showed that the highest strain values were localized in the region of microvoid nucleation, which corresponded spatially to the hydrostatic stress hot-spot predicted by the finite element model. Typically, engineers are not interested in fragments of fibers with aspect ratios close to one because they contribute almost no load bearing capability when compared to their longer cylindrical neighbors present in the microstructure^28–30^. Therefore, techniques and algorithms to detect and characterize fibers are typically not suitable for $l/d\le 1$, which can be easily confused with noise^30,31^. In this work, the fragment of glass was manually observed in the *in-situ* tomography images, detected as a fragment of a fiber, included in the microstructural simulation, and proved to be a region of high hydrostatic stress and a region of corresponding microvoid nucleation, as was seen in Fig. 3A,B, and identified more closely in Fig. 3C. This provides evidence that while short fragments (even as short as $l/d=1$) in the presence of long fibers can be ignored in elastic stiffness predictions, as they do not strongly influence the elastic properties, they can strongly influence damage initiation past the elastic regime and therefore must be included and considered in strength predictions.

In general, this work coupled experiments and simulations to show that large porosity manufacturing defects do not consistently play a role in initiating and propagating damage, and that additional microstructural features, like T-type configurations and overlapping fiber tips, play a critical role in the microstructure’s overall resistance to damage initiation. The experiment, which tracked approximately 92,000 fibers, as well as manufacturing and damage induced porosity, identified the critical region of the material that led to failure. A high fidelity model (comprised of 44.5 million elements) was created of this sub-region, in which 368 fibers were instantiated. The results showed that the 5 experimentally observed locations of microvoid nucleation corresponded with the 5 highest regions of hydrostatic stress, which substantiates the use of the hydrostatic stress metric to explain and predict microvoid nucleation in the microstructure of fiber reinforced thermoplastic composites, proving a long standing hypothesis of the role of stress triaxiality in damage initiation for thermoplastic matrix composites. Lastly, this work showed that although small fragments of fibers, which are almost negligible in size compared to the average fiber length, can be ignored during elastic mechanical property predictions, they must be included in the damage initiation and strength predictions of these composites.

## Methods

### Experimental details

**Figure 5:**
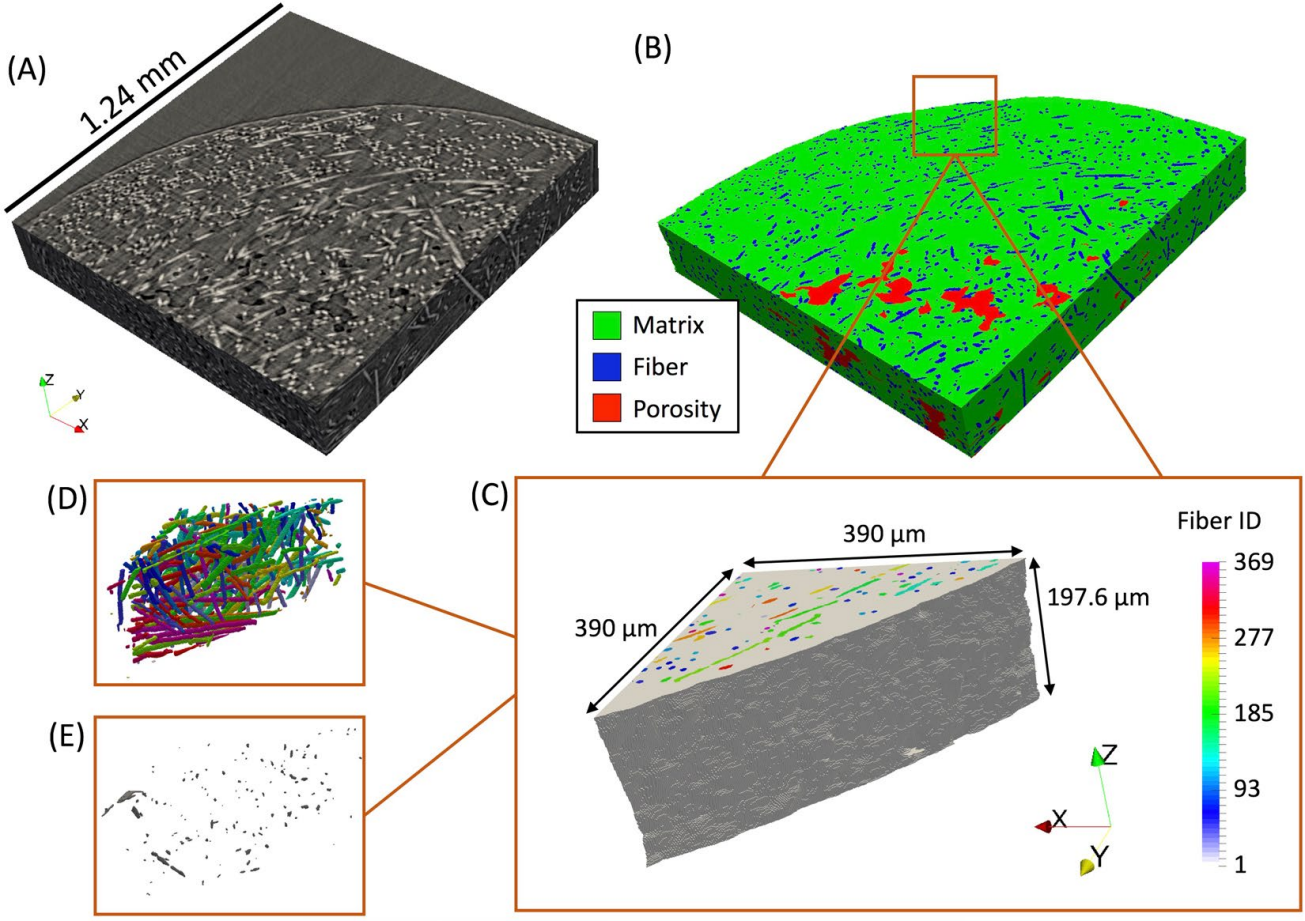
The sequence of post-processing the reconstructed tomography images in (**A**) to achieve high fidelity feature detection shown in (**B**), which includes specimen edge detection, porosity manufacturing defect detection, and individual fiber detection in 3D, as well as the instantiated model (**C**), the 368 fibers within the model (**D**), and the manufacturing porosity defects within the model (**E**).

The material used in this work was a polypropylene thermoplastic reinforced with 30% by weight E-glass fibers which were approximately 10 $\mu m$ in diameter and were pre-treated with a tailored silane solution to promote fiber-matrix adhesion. The composite material was injection molded into a cylindrical rod measuring 1.27 $cm$ in diameter and 45.72 $cm$ in length where the flow direction was in the length direction of the rod, and the rod was then machined into a dog-bone shaped specimen with a gauge section diameter of 2.4 $mm$ and length of 5 $mm$ containing fibers that were, on average, approximately 300 $\mu m$ long^31^. The specimen was studied *in-situ* by applying tensile load (at a quasi-static strain rate of approximately 0.001 ${s}^{-1}$) using a custom motorized screw driven miniature load frame, interrupting the tensile load by holding the cross-head displacement, and acquiring an *in-situ* X-ray $\mu$-CT scan. A total of 58 interruptions and scans were conducted from the unloaded state to final fracture which occurred at 5.77% strain. The experiments were conducted at Argonne National Laboratory using synchrotron X-rays with an X-ray energy of 25 $keV$, collected on an area detector placed 75 $mm$ downstream from the specimen. Each X-ray projection was acquired with a 100 $ms$ exposure time every 0.12$^\circ$ while the specimen and load frame were rotated at 0.5$^\circ$/$s$ through a 180$^\circ$ range. The use of synchrotron X-rays enabled a full $\mu$-CT scan to be acquired in 6 minutes. The 1500 X-ray projections captured at each scan were reconstructed using TomoPy^32^ resulting in 2D images which stack to form a 3D image with dimensions 2560 by 2560 by 1240 pixels, and a pixel size of 1.3 $\mu m$. The exterior surface of the specimen was painted to exhibit a black and white speckle pattern which was optically imaged at each tensile increment, in order to compute (using VIC-2D) the macroscopic strain experienced by the specimen. The reconstructed tomography images acquired just before failure were visually inspected in order to identify the region that contained the ductile fracture zone, which was traced back to the tomography images at the unloaded state shown in Fig. 5A using slice-by-slice 2D image correlation. The time lapse tomography images of the ductile failure zone were then inspected to identify the location of damage initiation, which was determined to be the region shown in Fig. 5C. The microscopic strains in the matrix at this region were computed using a DVC algorithm which makes use of fast Fourier transform based cross-correlation in conjunction with an iterative deformation method^33^ that was conducted on the tomography images at the unloaded state and at 30% of the macroscopic failure strain using a subset size of 64 pixels and a subset spacing of 4 pixels. In analyzing the DVC results, locations of high ${\varepsilon }_{zz}$ strain were defined as voxels with computed strain in the $99.{7}^{\mathrm{th}}$ percentile of the distribution of ${\varepsilon }_{zz}$. Finally, the tomography images at this region were further inspected in a 2D slice-by-slice comparison between the unloaded state, 30%, and 50% of the macroscopic failure strain to identify the locations of microvoid nucleation during damage initiation within the microstructure, which were then post-processed in 3D using the three detection approaches described below.

### Simulation details

Fibers were detected using an iterative and supervised 2D and 3D combined algorithm^22^. Porosity manufacturing defects (and in later loading steps, nucleation of microvoids) were detected using a combination of Weka machine learning segmentation^34^ and manual correction using ModLayer^35^. The specimen’s free surface was detected through 2D image processing of each tomography slice using an in-house MATLAB algorithm that utilized the intensity gradient at the free surface, which can be seen in Fig. 5A, and an initial guess of the approximate center and radius of the cylindrical specimen. This region was then adjusted by mapping the intensity values from a range of $[0,1]$ to a range of $[0.4,0.9]$, converted to a binary image using a threshold of 0.655 of the median intensity of the image (which accounts for X-ray energy fluctuations), dilated by a disk structural element with a radius of 4, and finally adjusted to fill any holes in the edge region. The detection of the fibers, pores, and the free surface of the quadrant containing the ductile fracture zone can be seen in Fig. 5B, with the simulated region’s free surface, fibers, and pores shown in Fig. 5C–E, respectively.

**Figure 6:**
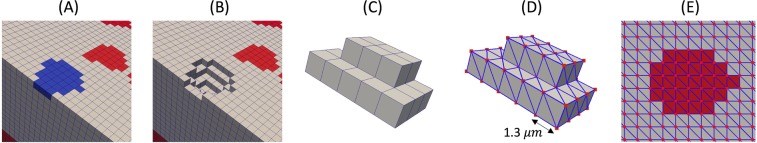
The procedure used to mesh the instantiated model where (**A**) shows a fiber in red, a porosity manufacturing defect in blue, and the matrix in grey, (**B**) shows the removed manufacturing pore, (**C**) an (**D**) show the voxels meshed using tetrahedral elements, and (**E**) shows a meshed fiber in the matrix.

The voxels (which were 1.3 $\mu m$ in size) corresponding to porosity manufacturing defects were removed from the rendered 3D volume as shown in Fig. 6A,B, and the remaining matrix and fiber features were meshed using tetrahedral elements directly from the voxelated microstructure shown in Fig. 6C,D. This procedure was conducted in ParaView, and resulted in uniform and ideal tetrahedral elements and perfect bonding between the fibers and the matrix, as shown in Fig. 6E, which can be generally assumed for fiber reinforced thermoplastics where the fibers have been pre-treated^11^. The meshed fiber elements were assigned linear elastic properties with an elastic modulus of 72.4 $GPa$ and a Poisson’s ratio of 0.2^27,36^. The meshed matrix elements were simulated to include nonlinear plasticity through a multilinear isotropic hardening model^37^. The positive X, negative Y, and negative Z surfaces were constrained using roller boundary conditions, and the positive Z surface was displaced in the positive Z direction by 7.8 $\mu m$, matching the displacement experimentally observed by the microstructure at 50% of the failure strain. The microstructural region shown in Fig. 5C, which was analyzed using finite elements, contained 7.56 million nodes and 44.5 million elements, and was solved using ABAQUS in 92.5 hours utilizing 300 processors and 1.92 $TB$ of memory. In analyzing the results of the simulation, hydrostatic stress hot-spots were defined as agglomerations of at least 1000 connected matrix elements that were all in the $99.99{7}^{\mathrm{th}}$ percentile of hydrostatic stress, slightly higher than the DVC analysis due to the finer mesh size.
